# Vergae cyst and autism spectrum disorder: a case report

**DOI:** 10.1192/j.eurpsy.2026.11120

**Published:** 2026-07-31

**Authors:** A. Souidi, Z. El Maataoui, H. Kisra

**Affiliations:** 1 Ar-Razi Hospital, Rabat, Morocco; 2 Child and adolescent psychiatry, Ar-Razi Hospital, Rabat, Morocco

## Abstract

**Introduction:**

The **cavum Vergae** is a fluid-filled space located posterior to the cavum septum pellucidum, between the bodies of the fornices. It results from the incomplete fusion of the two leaflets that form the septum pellucidum during embryonic development. When this space becomes enlarged, it is referred to as a **cavum Vergae cyst**—a rare, typically asymptomatic congenital lesion. However, when symptomatic, its clinical presentation can be variable and nonspecific. Midline cerebral anomalies such as cavum septum pellucidum, cavum Vergae, and agenesis of the corpus callosum have occasionally been reported in individuals with neurodevelopmental disorders, including ASD. However, studies exploring this association remain limited.

**Objectives:**

In this report, we describe the case of a child diagnosed with ASD in whom a cavum Vergae cyst was incidentally discovered on brain MRI. To our knowledge, this association has been rarely described, raising questions about the potential neurodevelopmental relevance of such midline anomalies

**Methods:**

A case report and litterature review.

**Results:**

We report the case of a 3-year-old boy referred for language delay noticed at the age of 2. He spoke his first words at 2.5 years. At daycare, he showed no social interaction with peers. There was no pointing, no spontaneous requests, no pretend play, and restricted interests, particularly in toy cars. Pregnancy was uneventful, with normal birth weight. The neonatal period was marked by jaundice requiring two days of phototherapy. Motor milestones were appropriate for age, and no neurological or genetic abnormalities were detected. The diagnosis of ASD was supported by standardized assessments: ADI-R (scores A:10, B:10, C:5, D:4) and ADOS-2 (total score: 20, comparison score: 6). EEG and auditory evoked potentials were unremarkable. Brain MRI revealed an isolated cavum Vergae cyst with no other structural abnormalities. The child is currently receiving behavioral therapy based on the ABA model and speech therapy, with notable clinical improvement.

**Conclusions:**

This case illustrates a rare co-occurrence of autism spectrum disorder and a cavum Vergae cyst, an association that remains scarcely reported in the literature. While the presence of such midline anomalies may be incidental, their potential role in neurodevelopmental processes warrants attention. Reporting such cases may help build a body of evidence to better understand the clinical significance of midline cerebral anomalies in children with ASD.

**Disclosure of Interest:**

None Declared

